# Resistance to mTOR Kinase Inhibitors in Lymphoma Cells Lacking 4EBP1

**DOI:** 10.1371/journal.pone.0088865

**Published:** 2014-02-21

**Authors:** Sharmila Mallya, Briana A. Fitch, J. Scott Lee, Lomon So, Matthew R. Janes, David A. Fruman

**Affiliations:** Department of Molecular Biology & Biochemistry, and Institute for Immunology, University of California Irvine, Irvine, California United States of America; University of Pittsburgh, United States of America

## Abstract

Inhibitors of the mechanistic target of rapamycin (mTOR) hold promise for treatment of hematological malignancies. Analogs of the allosteric mTOR inhibitor rapamycin are approved for mantle cell lymphoma but have limited efficacy in other blood cancers. ATP-competitive “active-site” mTOR inhibitors produce more complete mTOR inhibition and are more effective than rapamycin in preclinical models of leukemia, lymphoma and multiple myeloma. In parallel to clinical trials of active-site mTOR inhibitors, it will be important to identify resistance mechanisms that might limit drug efficacy in certain patients. From a panel of diffuse large B-cell lymphoma cell lines, we found that the VAL cell line is particularly resistant to apoptosis in the presence of active-site mTOR inhibitors. Mechanistic investigation showed that VAL does not express eukaryotic initiation factor 4E-binding protein-1 (4EBP1), a key negative regulator of translation controlled by mTOR. Although VAL cells express the related protein 4EBP2, mTOR inhibitor treatment fails to displace eukaryotic initiation factor 4G from the mRNA cap-binding complex. Knockdown of eukaryotic initiation factor 4E, or re-expression of 4EBP1, sensitizes cells to apoptosis when treated with active-site mTOR inhibitors. These findings provide a naturally occurring example of 4EBP deficiency driving lymphoma cell resistance to active-site mTOR inhibitors.

## Introduction

In order to maintain rapid proliferation and survival, cancer cells depend on high rates of protein synthesis and on selective translation of cap-dependent mRNAs encoding cell cycle regulators and anti-apoptotic proteins [1], [2]. Eukaryotic initiation factor 4E (eIF4E), which together with eukaryotic initiation factor 4G (eIF4G) and eukaryotic initiation factor 4A (eIF4A) form the cap-binding complex, is frequently overexpressed in human cancer and can cooperate with the Myc oncogene in an experimental lymphoma model [3]. Consequently, drugs targeting eIF4E and other translation factors have received increased attention as possible therapeutic approaches in leukemia and lymphoma [1], [4].

A key upstream regulator of eIF4E is the serine/threonine kinase mTOR [5]–[7]. Elevated mTOR activity is a prominent feature of cancer cells, including hematological malignancies [8]. The mTOR enzyme forms two complexes, TORC1 and TORC2, which are independently regulated and have distinct substrates. One set of important TORC1 substrates are the eIF4E-binding proteins (4EBPs), 4EBP1 and 4EBP2 [9]. When dephosphorylated, these proteins suppress cap-dependent translation by sequestering eIF4E. TORC1 phosphorylates 4EBPs to relieve eIF4E inhibition and promote cap-dependent translation.

The classical mTOR inhibitor rapamycin functions through an allosteric mechanism [10]. Rapamycin or its analogs (rapalogs) form an intracellular gain-of-function complex with FK506 binding protein 12 (FKBP12) that disrupts the stability of TORC1 and reduces phosphorylation of certain substrates. Rapalogs inhibit phosphorylation of S6 kinase (S6K) very efficiently, but have lesser impact on the phosphorylation of 4EBP1 and 4EBP2 by TORC1 [11].

Active-site mTOR inhibitors (asTORi) are a novel class of anti-cancer drugs that suppress both rapamycin-sensitive and rapamycin-resistant functions of TORC1 and TORC2 [8], [12]–[14]. In preclinical models of cancer, asTORi produce a stronger cytostatic response than rapamycin and can induce apoptosis especially when combined with other agents. The greater biological effects of asTORi relative to rapamycin have been linked to differential effects on the 4EBP-eIF4E axis. Supporting this correlation, recent studies have shown that reducing the ratio of 4EBP to eIF4E expression in experimental cell lines can increase sensitivity to asTORi [15], [16].

Diffuse large B-cell lymphoma (DLBCL) is a common hematological malignancy for which new therapeutic strategies are needed [17]. Targeting mTOR with asTORi represents a potential new approach. Here we report the discovery of a DLBCL line, VAL, which is intrinsically resistant to asTORi and lacks detectable expression of 4EBP1 mRNA or protein. 4EBP2 is expressed in VAL cells but does not block formation of the cap-binding complex following mTOR inhibition. In accord, asTORi fail to inhibit expression of a cap-dependent reporter plasmid and have minimal effects on protein synthesis in VAL cells. Knockdown of eIF4E or expression of 4EBP1 sensitizes VAL cells to asTORi. Low expression of 4EBP1 in a primary human DLBCL specimen was reported in a microarray study, and eIF4E overexpression is quite common (see Discussion). Our data suggest that low 4EBP1 expression and/or high eIF4E expression might be negative predictive markers for asTORi efficacy in lymphoma.

## Results

### Screening a Panel of Lymphoma Cell Lines for Sensitivity to MLN0128

Previous work in our lab established the efficacy of asTORi in models of pre-B acute lymphoblastic leukemia and demonstrated reduced hematotoxicity and immunosuppression compared to rapamycin or dual PI3K/mTOR inhibitors [18], [19]. These findings prompted us to test the effects of asTORi on more common human blood cancers such as DLBCL. This disease encompasses several subtypes of mature B cell lymphomas and is usually treated with combination chemotherapy plus anti-CD20 monoclonal antibodies [17]. Despite improvements in overall survival of DLBCL patients, new treatment options are needed to prevent and/or treat relapse. Several studies have shown growth-suppressive effects of rapamycin, PI3K inhibitors or dual PI3K/mTOR inhibitors in B lymphoma cell lines [20]–[22]. However, the effects of selective mTOR kinase inhibitors (asTORi) on DLBCL have not been reported.

**Figure 1:**
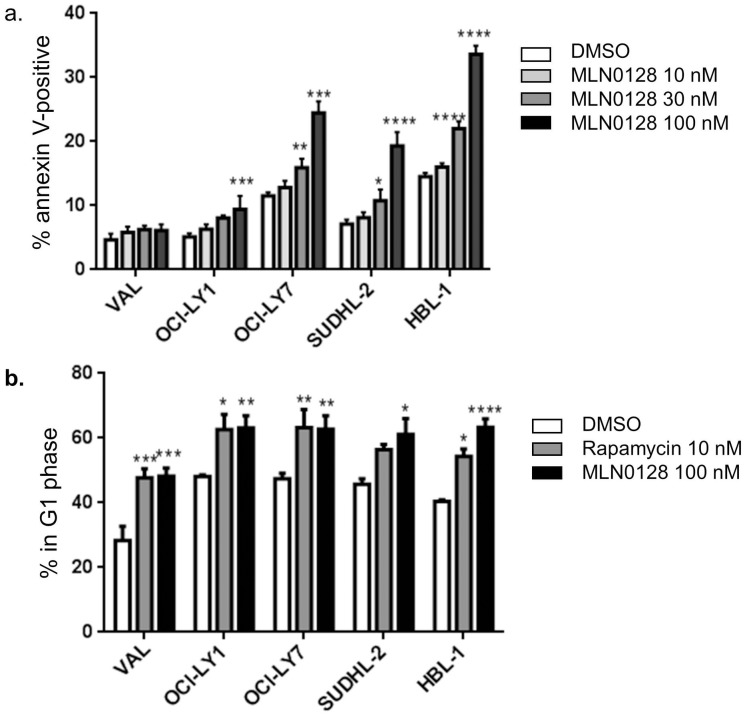
Differential sensitivity of DLBCL cell lines to asTORi. a. Annexin V and propidium iodide staining to assess DLBCL cell lines undergoing apoptosis following a 48* p<0.05, ** p<0.01, ***p<0.001, ****p<0.0001. b. Cell cycle analysis after a 48 hour treatment with the indicated inhibitors. Results graphed as a percentage of cells in G1 phase. n = 3, error bars represent SEM. * p<0.05, ** p<0.01, ***p<0.001, ****p<0.0001.

For most of the experiments in this study, we used the asTORi compound MLN0128 (formerly INK128) that is in clinical trials [19], [23], [24]. As we observed in pre-B leukemia cell lines [19], MLN0128 suppressed growth of DLBCL lines at concentrations 5–10 times lower than the first generation asTORi PP242 (**Fig. S1**). Next we tested the ability of MLN0128 to induce cell death in 5 DLBCL lines. Cells were treated with increasing concentrations of MLN0128 ranging from 10 nM to 100 nM for 48 hours, and the percentage of cells undergoing apoptosis was assessed by staining with Annexin V and propidium iodide. Three cell lines (HBL-1, OCI-LY7 and SUDHL-2) all showed a significant increase in apoptosis when treated with 30 nM or 100 nM MNL0128 (**Fig. 1a**). In the OCI-LY1 cell line, apoptosis increased significantly with 100 nM MLN0128 (**Fig. 1a**). In contrast, the VAL cell line showed no increase in cell death even at 100 nM MLN0128 (**Fig. 1a**). When we treated the DLBCL lines with a structurally distinct asTORi compound, AZD8055, we observed comparable effects with VAL cells again showing resistance to cell death (**Fig. S2**).

Cell cycle analysis with propidium iodide staining showed that MLN0128 had cytostatic effects in all cell lines tested, with an increased fraction of G1 phase (**Fig. 1b**) and decreased percentage in S and G2 phases (data not shown). The cytostatic effect was equivalent or slightly stronger than achieved by rapamycin.

In summary, our initial screen showed that DLBCL lines have a wide range of apoptotic sensitivity to asTORi, but all show cell cycle arrest or delay following drug treatment.

### The asTORi Resistant VAL Cell Line Lacks 4EBP1

**Figure 2:**
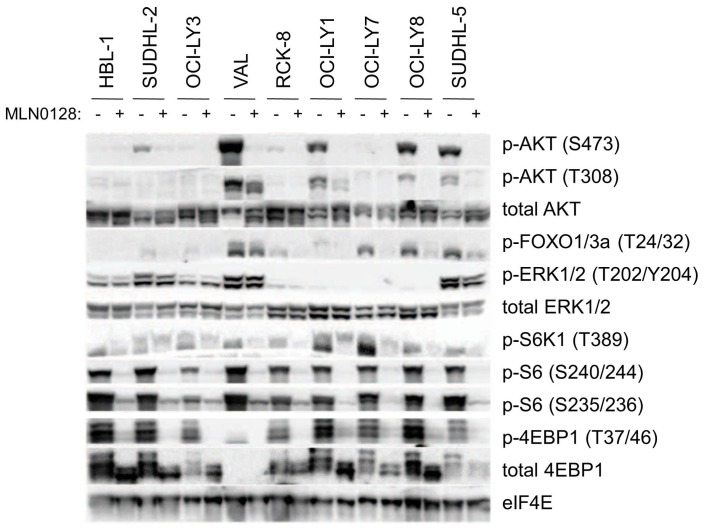
VAL cells lack 4EBP1. Western blot analysis of total cell lysates from DLBCL cell lines after a 4

**Figure 3:**
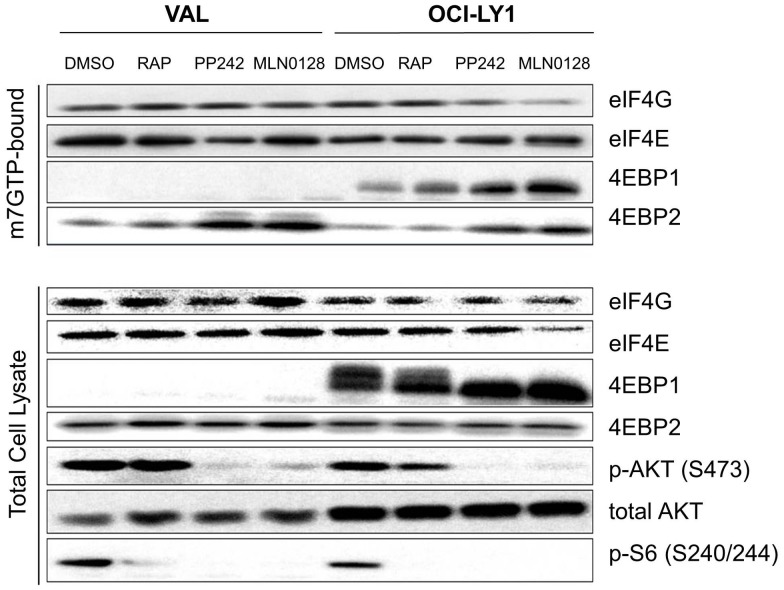
VAL cells are resistant to disruption of the translationally active eIF4F complex by asTORi. m^7^-GTP pull down assays of VAL and OCI-LY1 cells treated with 100 nM MLN0128, 500 nM PP242 or 10 nM rapamycin for 4 hours. Proteins from the cap bound fraction were detected by western blotting along with the total cellular proteins. Results are representative of several independent experiments.

The differences in sensitivity of DLBCL cell lines to asTORi led us to check for any differences in the signaling profiles upon mTOR inhibition. Using a broader panel of 9 DLBCL lines, we found that 200 nM MLN0128 effectively inhibited phosphorylation of both TORC1 (phospho-S6, phospho-4EBP1) and TORC2 (phospho-AKT-S473) outputs in all the cell lines tested (**Fig. 2**). When VAL cells were treated with the 100 nM concentration of MLN0128 used in the cell death assays, TORC1 and TORC2 outputs were still fully suppressed (**Fig. 3**). This suggests that differential sensitivity to asTORi is not due to resistance at the level of mTOR activity. Furthermore, we did not find evidence for 4EBP1 phosphorylation that is resistant to asTORi, a phenomenon observed in *KRAS*-mutant colon cancer cells [25].

An interesting observation from these western blots was that the VAL cell line did not express any 4EBP1 protein (**Fig. 2** **,** **3**). However, 4EBP2 was expressed (**Fig. 3**). To determine whether 4EBP1 expression in VAL cells was also reduced at the transcriptional level, we measured 4EBP1 mRNA using a two-step RT-PCR and β-actin as an internal control. There was no detectable 4EBP1 mRNA in VAL cells in contrast to the OCI-LY1 and OCI-LY7 cell lines (**Fig. S3**). All three cell lines had detectable 4EBP2 mRNA expression (**Fig. S3**).

Because TORC1 activity prevents 4EBPs from displacing eIF4G binding to eIF4E in the cap binding complex, we wanted to see if the absence of 4EBP1 in VAL cells maintained the active complex (also known as the eIF4F complex) upon MLN0128 treatment. In studies of Ph+ B-ALL cell lines we found that 100 nM MLN0128 treatment for 4 hours was effective at inhibiting the formation of cap binding complex without affecting cell viability or inducing off target effects (data not shown). Using m^7^-GTP pull down assays, we observed that a 4 hour asTORi treatment with MLN028 or PP242 did not reduce the amount of eIF4G bound to eIF4E (**Fig. 3**). This contrasted with the 4EBP1-expressing control cell line, OCI-LY1, in which treatment with MLN0128 or PP242 decreased the bound eIF4G along with a corresponding increase in 4EBP1 binding to eIF4E. Rapamycin caused a modest increase in 4EBP1 binding to eIF4E in OCI-LY1 cells, but little displacement of eIF4G. We also observed that VAL cells expressed the isoform 4EBP2, and that asTORi treatment did increase the amount of 4EBP2 bound to the cap complex. Nevertheless, the induced binding of 4EBP2 to eIF4E seemed to be ineffective at displacing eIF4G and was therefore unable to compensate for loss of 4EBP1. Blotting for total eIF4E was used as a control to confirm equal pulldown in the untreated and asTORi treated samples, and additional blotting of the total cell lysates confirmed inhibition of TORC1 and TORC2 substrate phosphorylation by asTORi. These results suggest that asTORi treatment in the VAL cells is ineffective at inhibiting the formation of the eIF4F translation initiation complex.

### MLN0128 Minimally Affects Cap Dependent Translation and Overall Protein Synthesis in VAL Cells

**Figure 4:**
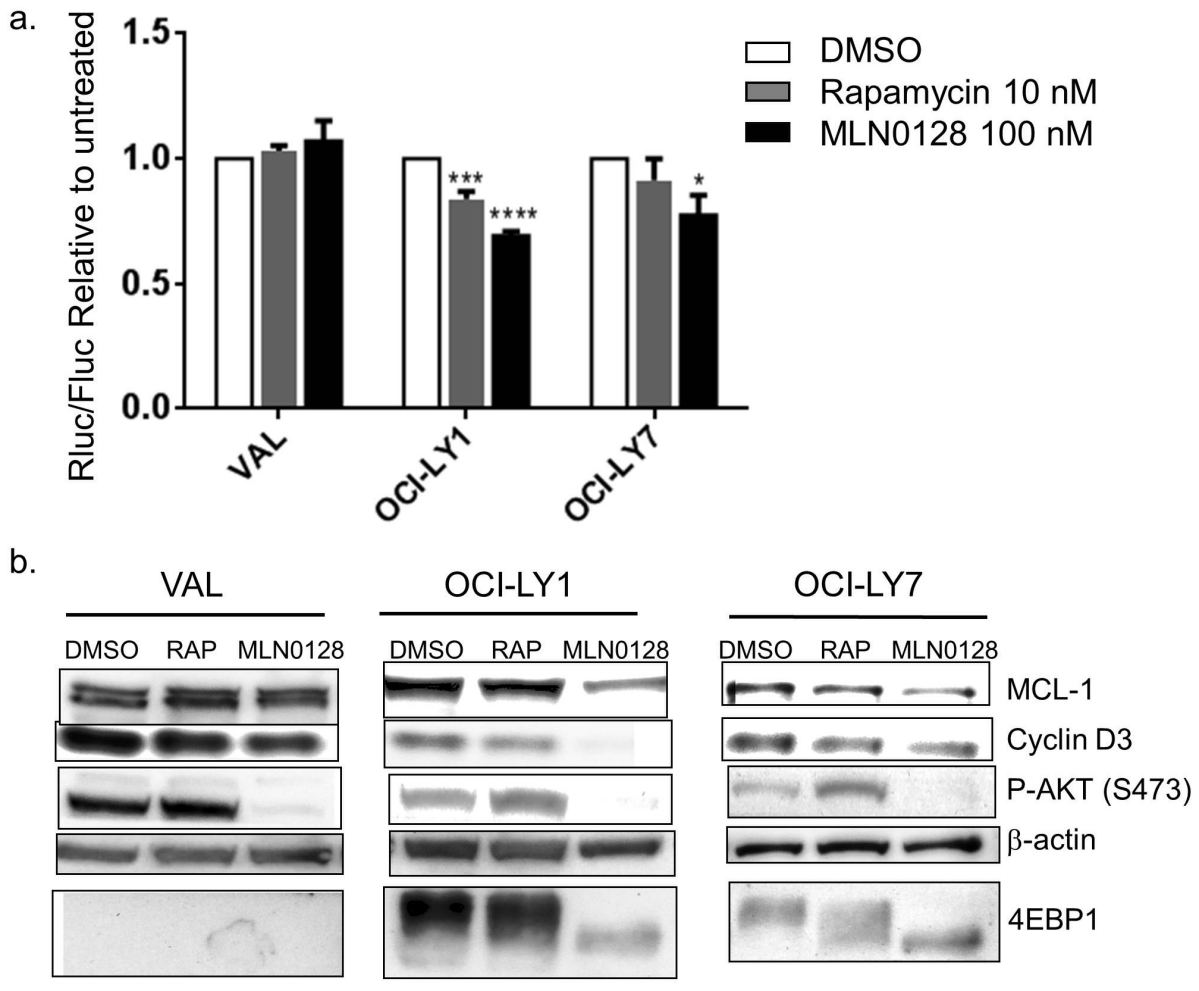
VAL cells maintain cap dependent translation and MCL-1 expression following asTORi treatment. a. A dual luciferase reporter construct was electroporated in the VAL, OCI-LY1 and OCI-LY7 cell lines followed by a 16 hr treatment with 100 nM MLN0128 or 10 nM rapamycin. Cap dependent renilla luciferase expression was measured relative to cap independent firefly luciferase readouts. Results were normalized to untreated control and averaged for 4 independent experiments. Statistical significance measured using ANOVA (mean +/− SEM, * p<0.05, ***p<0.001, ****p<0.0001). b. Western blot analysis of total cell lysates to assess MCL-1 and Cyclin D3 protein levels in VAL, OCI-LY1 and OCI-LY7 treated with 100 nM MLN0128 for 4 hours. Cells were partially synchronized by serum starvation in 0.1% FBS media for 24 hours followed by addition of 10% FBS along with the inhibitors for 4 hours. Results representative of 3 independent experiments.

We next tested whether maintenance of the eIF4F complex in VAL cells treated with MLN0128 preserved cap dependent translation and overall protein synthesis. First we used a dual luciferase reporter construct (pRSTF-CVB3) containing a cap independent firefly luciferase downstream of the 5 ' UTR of coxsackie virus B3 and an upstream cap dependent Renilla luciferase. Following transfection and a treatment with 10 nM rapamycin or 100 nM MLN0128, cell extracts were prepared after 16 hours to quantify peak renilla and firefly luciferase expression. Renilla luciferase activity was normalized to the cap independent firefly luciferase activity. Strikingly, mTOR inhibition did not decrease cap dependent translation in the VAL cells relative to the untreated controls (**Fig. 4a**). In contrast, the 4EBP1-expressing control cell lines OCI-LY1 and OCI-LY7 showed a significant ∼30% decrease in cap dependent translation with MLN0128, with an intermediate effect of rapamycin consistent with the lesser effect of rapamycin in the cap binding assay. Analysis of the raw luciferase values showed that MLN0128 caused a greater decrease in Renilla luciferase expression in the OCI-LY1 and OCI-LY7 cells compared to the VAL cells, while the decreases in firefly expression were smaller and comparable between the three cell lines (**Fig. S4**).

We also assessed whether maintenance of cap dependent translation upon mTOR inhibition in VAL cells correlates with protein levels of cap dependent transcripts like MCL-1 and Cyclin D3. Indeed, MLN0128 and rapamycin did not decrease MCL-1 protein amounts in VAL cells, in contrast to the 4EBP1 expressing cell lines OCI-LY1 and OCI-LY7 (**Fig. 4b**). Cyclin D3 protein decreased to varying degrees in all cell lines with both rapamycin and MLN0128 treatment. This is consistent with the cytostatic effects of MLN0128 observed in all three cell lines. Quantitative RT-PCR showed no significant differences in mRNA expression for MCL-1 or cyclin D3 at these time points (**Fig. S5**), supporting the conclusion that mTOR inhibition decreases MCL-1 protein in the OCI-LY1 and OCI-LY7 cell lines at the translational level.

**Figure 5:**
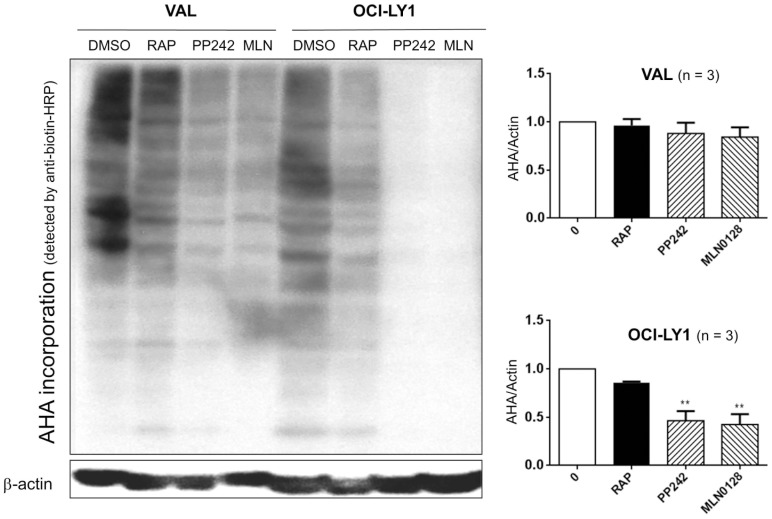
Protein synthesis in VAL cells is resistant to mTOR inhibition. Nascent protein synthesis was analyzed by measuring the uptake of L-azidohomoalanine (AHA). VAL and OCI-LY1 cells were treated with 100 nM MLN0128, 500 nM PP242 and 10 nM Rapamycin for 4 hrs followed by labeling with AHA for 2 hrs. Cells were lysed and labeled proteins were detected using a click-it chemistry based reaction. The incorporated AHA was detected using a western blot with anti-biotin HRP conjugated antibody. The signal intensity for biotin was normalized to actin (a long-lived protein reference) and results graphed relative to untreated control (right panel). n = 3, error bars represent SEM, ** p<0.01.

Next we assessed total protein synthesis using a non-radioactive assay that measures uptake of an azide-linked methionine (L-azidohomoalanine, AHA) by cells during nascent protein synthesis. The incorporated AHA was labeled with biotin using a Click-IT chemistry based reaction, followed by a western blot with anti-biotin HRP. In control OCI-LY1 cells MLN0128 caused a profound inhibition of protein synthesis, with intermediate effects of rapamycin (**Fig. 5**). The compound PP242 that is structurally related but ∼ 5–10-fold less potent than MLN0128 (see Fig. S1 and references ([19], [24]) had similar effects when added at 500 nM. In comparison, asTORi or rapamycin caused much less suppression of total protein synthesis in VAL cells. Thus, mTOR inhibition in VAL cells is ineffective at decreasing cap dependent translation and has negligible effects on overall protein synthesis.

### eIF4E Knockdown in VAL Cells Sensitizes them to asTORi

**Figure 6:**
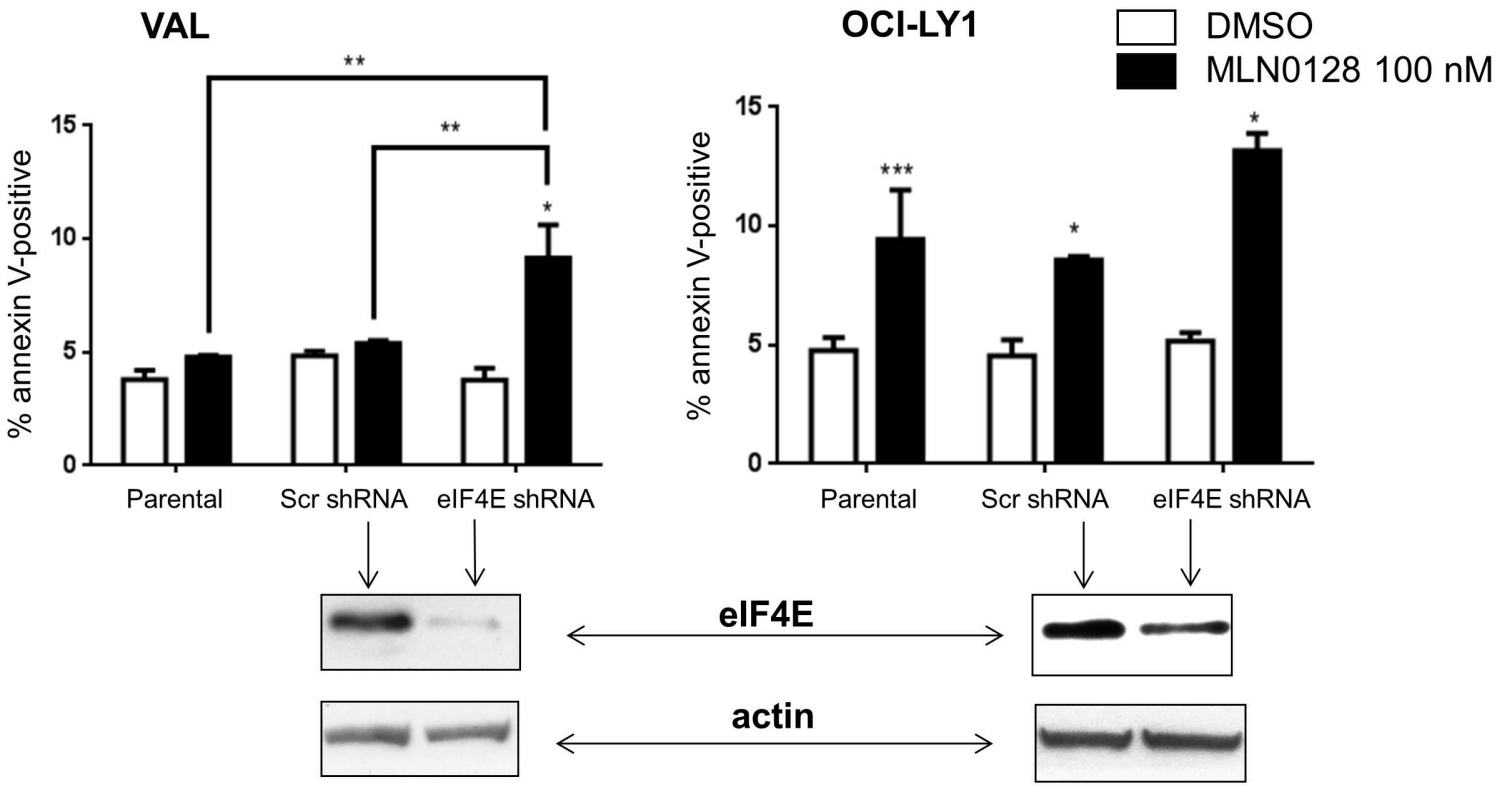
Knockdown of eIF4E increases VAL cell sensitivity to MLN0128. VAL and OCI-LY1 cells stably expressing eIF4E shRNA or scrambled shRNA control were treated for 48 hours with 100 nM MLN0128. Cell death was measured by Annexin V and PI staining. Results represent the percentage of cells that are Annexin V positive only and are averaged for 3 different experiments. Statistical significance was measured using a student’s t-test (paired, two-tailed) with error bars representing SEM (*p<0.05, **p<0.01 ***p<0.001,****p<0.0001). Shown below the graphs are western blots depicting the efficiency of eIF4E knockdown compared to the scrambled shRNA controls.

The results above suggested that maintenance of cap dependent translation following mTOR inhibition might play a pivotal role in the resistance of VAL cells to asTORi. This led us to test whether modulating the stoichiometry of the cap translation complex would sensitize VAL cells to asTORi treatment. Our first approach was to achieve knockdown of eIF4E in VAL cells (**Fig. 6**). Knocking down eIF4E did not affect basal survival or proliferation of VAL cells but increased sensitivity to cell death following MLN0128 treatment (**Fig. 6**). The parental and scrambled-shRNA control VAL cells behaved as expected with no significant increase in death with MLN0128. In OCI-LY1 cells that are basally sensitive to inhibition of cap dependent translation by asTORi, knocking down eIF4E did not significantly augment the cell death response compared to the scrambled-shRNA control (**Fig. 6**). The increase in asTORi sensitivity of VAL cells with eIF4E knockdown was corroborated when apoptosis was measured by sub-diploid DNA content (****).

A cap binding assay suggested that eIF4E knockdown in VAL cells did correlate with eIF4G displacement following MLN0128 treatment; however, firm conclusions were difficult due to the low signal of eIF4E and associated proteins in the knockdown cells (data not shown). VAL cells with eIF4E knockdown did show a slight decrease in MCL-1 protein levels upon asTORi treatment (**Fig. S7**). Overall, the results show that it is possible to increase VAL cell sensitivity to asTORi by reducing the amount of the cap binding protein eIF4E.

### Adding Back 4EBP1 in VAL Cells Increases Sensitivity to asTORi

**Figure 7:**
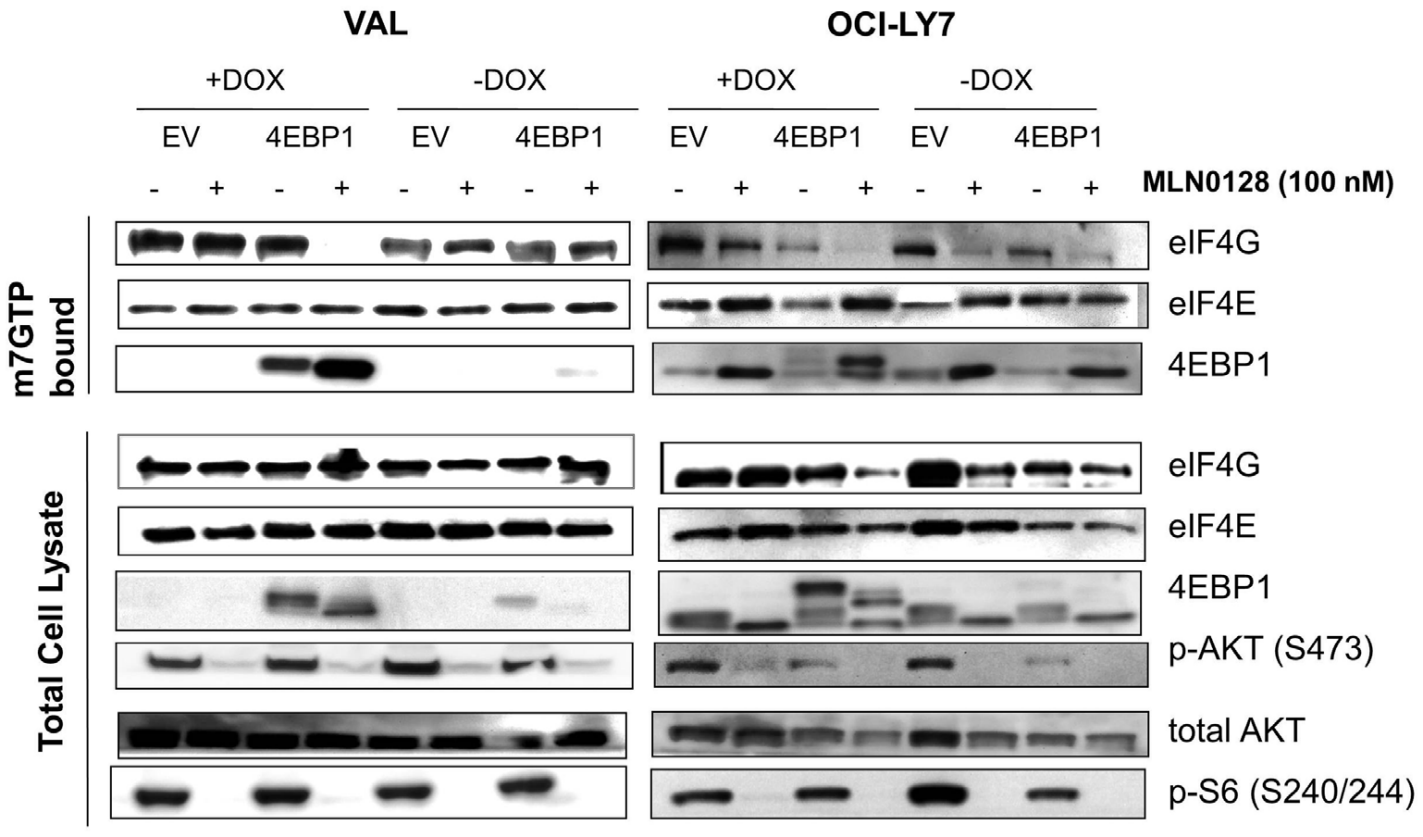
Expressing 4EBP1 in the VAL cells restores the effects of asTORi on cap dependent translation. m^7^-GTP pull down assay to measure the levels of cap bound proteins after a 4 hour MLN0128 treatment in the VAL and OCI-LY7 cells expressing 4EBP1 or the empty vector (EV) control. 4EBP1 expression was induced with 1 µg/ml Doxycycline for 16 hrs followed by a 4 hr treatment with 100 nM MLN0128. Results are representative of two independent experiments.

We next tested whether expressing 4EBP1 in VAL cells would yield similar results as eIF4E knockdown. Using a doxycycline-inducible system, we expressed 4EBP1 in VAL cells and in the cell line OCI-LY7 that is highly sensitive to asTORi. Analysis of the cap binding complex with a m^7^-GTP pull down assay showed that in VAL cells with 4EBP1 “add-back”, asTORi treatment increased the amount of 4EBP1 binding to eIF4E and concomitantly decreased the eIF4G association (**Fig. 7**). The empty vector control cells behaved similarly to the parental VAL cells in that MLN0128 treatment did not affect eIF4F formation, correlating with absence of 4EBP1. In the asTORi-sensitive OCI-LY7 cells where MLN0128 basally affects formation of the eIF4F complex, expression of 4EBP1 did not have any apparent effects.

**Figure 8:**
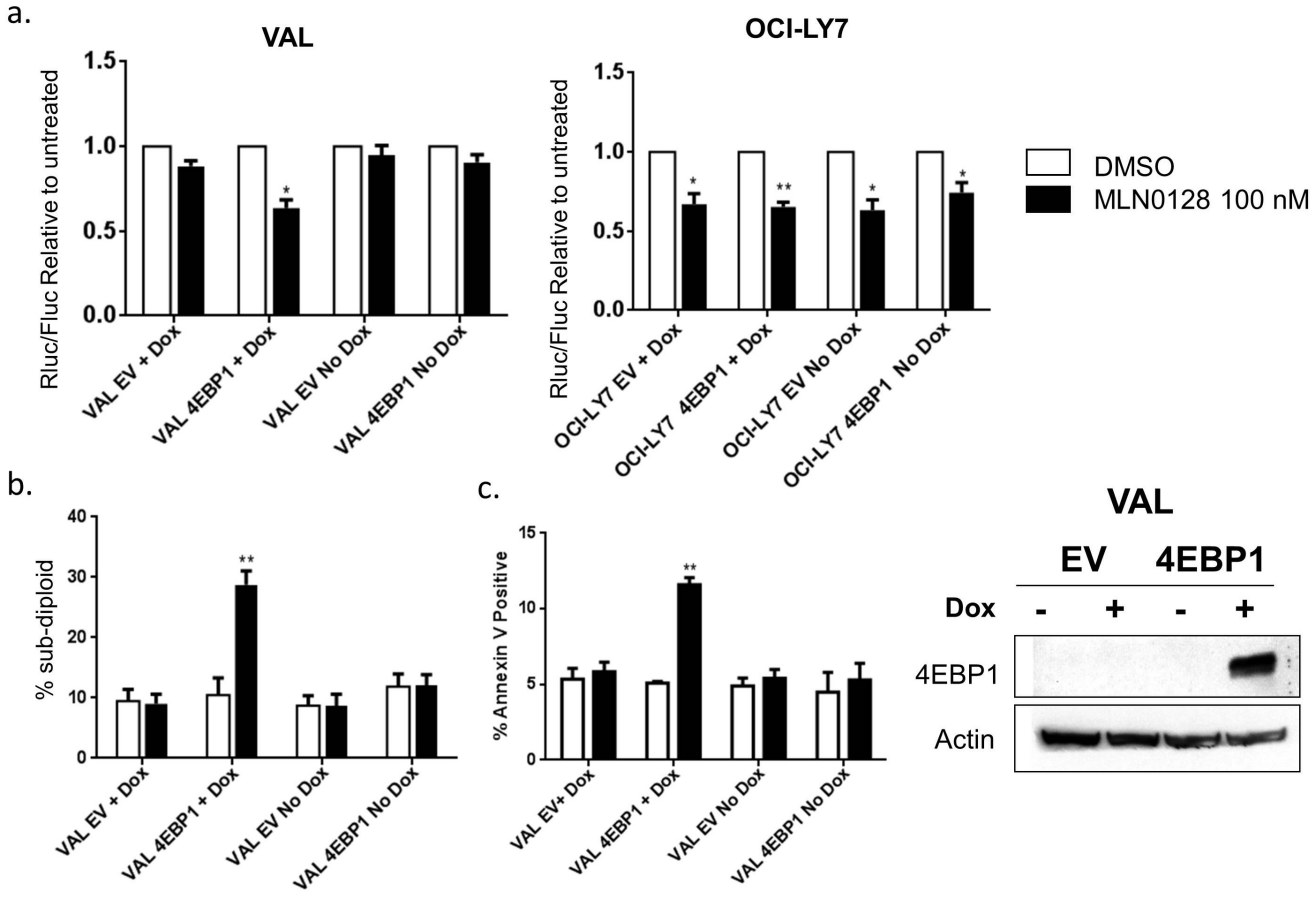
4EBP1 expression in the VAL cells causes asTORi mediated decrease in cap dependent translation and sensitizes them to MLN0128. a. Luciferase reporter assays in VAL and OCI-LY7 cells expressing 4EBP1. Cells were electroporated with the dual luciferase reporter constructs and treated with 100 nM MLN0128 (+/−1 µg/ml Doxycycline) for 16 hrs. Results are averaged for three independent experiments and statistical significance measured using a t-test (*p<0.05, **p<0.01). b. Propidium iodide staining to measure the percentage of cells with sub-diploid DNA content in the VAL cells expressing 4EBP1 upon treatment with 100 nM MLN0128 with and without doxycycline induction. Results averaged for three independent experiments. Statistical analysis using a t-test (**p<0.01). c. Annexin V and PI staining to measure the percentage of cells undergoing apoptosis in the VAL cells expressing 4EBP1 upon treatment with 100 nM MLN0128 with and without doxycycline induction for 48 hours. Results averaged for three independent experiments. Statistical analysis using a t-test (**p<0.01). Shown to the right of the graphs is a western blot showing induction of 4EBP1 expression with 1 µg/ml Doxycycline.

Next we wanted to test whether asTORi treatment in 4EBP1 add-back VAL cells decreased cap dependent translation. Indeed, as measured by dual luciferase reporter assays, the 4EBP1 add-back VAL cells showed a 30% decrease in cap dependent translation upon treatment with MLN0128 when compared to the untreated controls (**Fig. 8a**). This decrease was similar in magnitude to the effect seen in OCI-LY1 control cells (Fig. 4a). The VAL cells expressing empty vector and VAL cells with the 4EBP1 construct but without doxycycline induction behaved similarly to the parental VAL cells with no decrease in cap dependent translation following MLN0128 treatment (**Fig. 8a**). As observed in the cap-binding assay, overexpression of 4EBP1 in OCI-LY7 did not alter the sensitivity to MLN0128.

Lastly we assessed whether induced 4EBP1 expression in VAL cells potentiated a cell death response to MLN0128 treatment. Using propidium iodide staining to measure the percentage of cells in sub-G1 phase, we observed 30% death following MLN0128 treatment of 4EBP1 add-back VAL cells compared to the various controls (**Fig. 8b**). Annexin V and PI staining was used to confirm these results where VAL cells with 4EBP1 expression showed a 2-fold increase in Annexin V positive population when treated with MLN0128 (**Fig. 8c**). We observed a similar increase in sensitivity when the cells were treated with a structurally distinct asTORi, AZD8055, indicating that these results are specific to mTOR inhibition (data not shown). In summary, add-back experiments supported the conclusion that absence of 4EBP1 is one of the contributing mechanisms of asTORi resistance in VAL cells.

## Discussion

In this study we have identified a human DLBCL line whose resistance to asTORi can be attributed in part to a low ratio of 4EBP/eIF4E expression. VAL cells lack protein expression of 4EBP1, a key inhibitor of eIF4F formation. Although the cells express 4EBP2, treatment with asTORi fails to displace eIF4G from eIF4E and causes minimal inhibition of cap-dependent translation and protein synthesis. Knocking down eIF4E or adding back 4EBP1 can help circumvent the resistance to asTORi, sensitizing cap dependent translation and potentiating cell death. Our results agree with recent findings that the 4EBP/eIF4E ratio is a crucial determinant of asTORi sensitivity in cancer cells [15], [16], and extend this model to a naturally occurring DLBCL line deficient in 4EBP1.

Our data suggest that reduced 4EBP expression might be a biomarker of resistance to asTORi and to dual PI3K/mTOR inhibitors in DLBCL and other blood cancers. Of note, the OCI-LY3, RCK-8 and SU-DHL5 cell lines had reduced amounts of 4EBP1 compared to other cell lines (Fig. 2) and were mostly resistant to asTORi-induced death (data not shown). However, asTORi still reduced cap dependent translation in these cell lines as judged by reporter assays (data not shown). We focused our additional studies on VAL cells in which 4EBP1 mRNA and protein were absent and cap dependent translation was resistant to asTORi. Although we could readily detect 4EBP2 protein in cell lysates and cap-binding assays, the presence of this isoform is not sufficient to confer asTORi sensitivity in VAL cells lacking 4EBP1. We cloned and sequenced the cDNAs for eIF4F components (eIF4A, eIF4E, eIF4G) expressed in VAL cells and did not identify any mutations that might explain the preservation of eIF4G binding upon asTORi-induced recruitment of 4EBP2 (data not shown). We did not measure expression of the third isoform, 4EBP3, but if it is present in VAL cells it also cannot substitute for 4EBP1.

If eIF4E expression is in excess of eIF4G and 4EBP2 in VAL cells, this might explain why asTORi-triggered 4EBP2 recruitment does not affect eIF4G binding. Consistent with this model, eIF4E knockdown rendered VAL cells sensitive to asTORi effects on cap-dependent translation and cell death. In VAL cells with eIF4E knockdown, MLN0128 reduced MCL-1 expression and this might contribute to the increased apoptosis.

In addition to conferring mTOR inhibitor resistance, a reduced 4EBP:eIF4E ratio might help to drive the tumor phenotype by facilitating translation of oncogenic mRNAs. eIF4E overexpression has been noted in many cancer types [2], [4], [6], and eIF4E overexpression in a mouse model cooperated with Myc to cause B cell transformation [3]. In accord, a search of the Oncomine database revealed frequent overexpression of eIF4E mRNA in Burkitt’s Lymphoma, a cancer driven by Myc (**Fig. S8**). The same gene array study (Basso Lymphoma) showed that a majority of primary DLBCL specimens express higher levels of eIF4E mRNA compared to normal B cells or centroblasts (**Fig. S8**). Notably, we were not able to achieve stable knockdown of eIF4E in some DLBCL cell lines (OCI-LY7 and SUDHL-4). The cells infected with eIF4E shRNA viruses did not grow out of selection compared to the scrambled shRNA controls (data not shown), supporting the idea that some DLBCLs depend on high levels of eIF4E for their survival.

We searched the Basso Lymphoma dataset for patterns of 4EBP1 expression. One primary DLBCL specimen (# GSM44245), out of 32 tested, had greatly reduced 4EBP1 mRNA expression relative to resting B lymphocytes and centroblasts (**Fig. S9**). Therefore, 4EBP1 loss might occur in a fraction of primary human DLBCL tumors as observed in the VAL cell line. eIF4E expression in sample GSM44245 was higher than in normal B cells and similar to centroblasts. Nine other subtypes of B cell leukemia or lymphoma were analyzed in this dataset and none showed evidence for loss of 4EBP1 expression. Considering that there are three members of the 4EBP family, their tumor suppressor functions might be redundant. Of note, a very recent study showed that a large fraction of human pancreatic cancers lose expression of 4EBP1 [16]. However, 4EBP2 expression was not detected in pancreatic cancers or cell lines, a phenomenon that might facilitate tumor progression following 4EBP1 loss. In all B cell malignancies tested in the Basso Lymphoma microarray, 4EBP2 expression was similar to normal B cells (**Fig. S10**).

Increasing the 4EBP:eIF4E ratio rendered VAL cells more sensitive to asTORi-induced apoptosis, yet the magnitude of the cell death was still limited. Similarly, the pro-apoptotic effects of MLN0128 and AZD8055 were modest in the DLBCL lines that expressed 4EBP1. These observations suggest that in isolation, mTOR kinase inhibition and disruption of eIF4F is generally insufficient to kill DLBCLs. In addition, there are likely to be other mechanisms of resistance to asTORi that are unrelated to the 4EBP:eIF4E ratio. Further investigations should focus on combination approaches that maximize the pro-apoptotic potential of mTOR inhibitors.

## Materials and Methods

### Reagents

MLN0128 was provided by Intellikine or purchased from Active Biochem (Maplewood, NJ). AZD8055 was purchased from Active Biochem, PP242 and Rapamycin from LC labs (Woburn, MA). All compounds were dissolved in DMSO at 10 mM and stored frozen in aliquots.

### Cell Lines and Cell Culture Conditions

DLBCL cell lines VAL, OCI-LY1, OCI-LY7, HBL-1, SUDHL-4, SUDHL-5, RCK-8, OCI-LY8, SUDHL-2 were obtained from Dr. Laura Pasqualucci (Columbia University, NY) who has described these cell lines previously [26]. We chose these cell lines from a larger panel of DLBCLs because they express BAX, BAK and BIM and are therefore have an intact apoptotic machinery. Cells were cultured in IMDM (Thermo Scientific Hyclone) with 10% fetal bovine serum (Omega scientific, Lawrenceville, GA), 1% HEPES, 1% Glutamine, 1% Pen-Strep (Mediatech Inc, Manassas, VA) in a humidified incubator with 5% CO_2_ at 37°C.

### Lentiviral shRNA Knockdown of eIF4E

The pLKO.1 lentiviral vectors with eIF4E and scrambled shRNA (Sigma Aldrich, St. Louis, MO) were used with pCMV-VSVG envelope (Addgene plasmid 8454,Weinberg lab) and psPAX2 packing plasmids (Addgene plasmid 12260, Trono lab) to generate infectious lentiviral particles. VAL and OCI-LY1 cell lines were infected with high titer lentivirus and selected with 2 µg/ml puromycin (Sigma Aldrich). The knockdown of eIF4E was confirmed by western blotting.

### Generation of Cell Lines with Inducible Expression of Wild Type 4EBP1

Full length rat wild type 4EBP1 was obtained from Dr. John Lawrence (University of Virginia, Charlottesville, VA). This construct was cloned into the pLVX-puro Dox inducible system (Clontech, Mountain View, CA) using Not1 and EcoR1 restriction sites. High titer lentivirus was produced using the lentiviral packing and envelope constructs mentioned above. VAL and OCI-LY7 cell lines expressing the tetracycline transactivator protein (rtTA) were infected with the lentivirus and selected in IMDM culture medium containing 8 µg/ml Blasticidin and 2.0 µg/ml Puromycin. The wildtype 4EBP1 expression was induced by addition of 1 µg/ml Doxycycline for 16–24 hrs.

### Western Blotting

Western analysis was performed as previously described [18]. The following antibodies were used for immunoblotting: eIF4E, 4E-BP1, eIF4G, 4EBP2, MCL-1, phospho-S6 (S240/244) (Cell Signaling Technology, Beverly, MA), actin (Sigma-Aldrich). Antibody dilutions were performed according to the manufacturer's instructions. Immunoreactive bands were visualized by enhanced chemiluminescence (GE Healthcare Life sciences, Pittsburgh, PA) after incubation with HRP conjugated secondary antibody (Promega, Madison, WI).

### Cap-binding Assay

Analysis of the cap-binding complex was performed by adapting a previously described protocol [27]. Following incubation with inhibitors, 4–5×10^6^ cells were lysed by three freeze–thaw cycles in freeze–thaw lysis buffer. In all, 100–200 µg of protein from the lysates was then incubated with a suspension of 7 methyl-GTP-Sepharose 4B beads (GE Healthcare Life sciences), and placed on a rocker at room temperature. After 1 h, the beads were pelleted and washed twice with lysis buffer. The beads were then boiled in running buffer for 5 min, separated by sodium dodecyl sulfate–polyacrylamide gel electrophoresis (SDS–PAGE), transferred to nitrocellulose membranes, and subjected to Western analysis.

### Cell Cycle and Cell Death Analysis

1×10^6^ cells were harvested, fixed in 50% ethanol, washed and stained in 0.1 mg/ml propidium iodide (PI)-staining solution (Life Technologies, Grand Island, NY). Cells were then analyzed on a FACSCalibur flow cytometer (Becton-Dickinson, San Jose, CA), and cell cycle analysis performed using FlowJo software v.5.7.2 (TreeStar, Ashland, OR).

After exclusion of cell debris, the induction of cell death was measured by calculating the percentage of intact cells with Sub-G1 DNA content. Cell death was also measured using Annexin V (Life Technologies, Grand Island, NY) and propidium iodide (PI) (Life Technologies, Grand Island, NY) staining.

### Tetrazolium-based Proliferation Assays

Cells (5×10^3^) were plated in triplicate with DMSO or inhibitors in a 100 µl volume in a 96-well plate at 37°C. After 48 or 72 h, CellTiter 96 AQueous One solution (Promega) was added to each well and processed per the manufacturer's instructions. The optical density for each condition was calculated as a percentage of the untreated control after subtracting for background absorbance.

### Measurement of Nascent Protein Synthesis

Cells were treated with inhibitors for 4 hr and then washed with PBS and resuspended in methionine free RPMI 1640 media (Life Technologies) with 1% FBS for 1 hour. The cells were labeled with 100 µM of the methionine analog, L-azidohomoalanine (AHA) (Cat # C10102, Life Technologies) for 2 hr followed by washing in PBS and lysing in lysis buffer (50 mM Tris HCl, pH 8.0, 1% SDS). The AHA labeled proteins were then detected using the Click-iT Biotin protein analysis Detection kit (Cat# C33372, Life Technologies) as per the instructions. The detected proteins were then separated on a SDS PAGE gel and transferred to a nitrocellulose membrane and probed with Anti-Biotin, HRP-linked antibody (Cat# 7075, Cell Signaling).

### Luciferase Reporter Assays to Measure Cap Dependent Translation

Luciferase reporter construct pRSTF-CVB3 containing the 5 ' NCR of the Coxsackie B3 virus [28]. cloned between a firefly and renilla luciferase was used to measure cap dependent translation. The construct was electroporated in a FBS free media and cells were allowed to recover in complete IMDM media for 2 hrs followed by inhibitor treatment for 16 hrs. Following treatment, cells were lysed and renilla and firefly luciferase expression was measured using the Dual luciferase assay kit (Promega) using a luminometer. Cap dependent renilla luciferase expression was normalized to cap independent firefly luciferase expression and results were expressed relative to untreated control.

### Quantitative Real Time PCR

RNA was extracted from cells using Trizol (Life Technologies). 1 µg of RNA was reverse transcribed using the iScript cDNA synthesis kit (Biorad, Hercules, CA). Gene specific primers were used to amplify MCL-1, Cyclin D3 and Actin using the Step One Real time PCR system and relative quantification of the transcripts was done using the delta delta cT method. 4EBP1, 4EBP2 and actin specific primers were used in a 30 cycle PCR reaction using the S1000 thermal cycler (Biorad) to check for expression of the relevant mRNA transcripts.

## Supporting Information

Figure S1 MTS assay to identify effective concentration ranges of mTOR inhibitors in DLBCL cell lines. Cells were treated with indicated concentrations of MLN0128, PP242, rapamycin or BEZ235 for 48 hours. Results were averaged from three independent experiments and plotted as percent growth inhibition. Error bars represent standard error of the mean (SEM).
(TIFF)

Figure S2 Differences in cell death among DLBCL lines was confirmed by staining with the vital dye 7-AAD. The percent increase in 7-AAD+ relative to DMSO control (which was 5–10%) is shown (n = 3 for VAL, OCI-LY1, HBL1; n = 1 for OCI-LY7 and SUDHL-2).
(TIFF)

Figure S3 RT-PCR to check for 4EBP1 mRNA expression in the VAL, OCI-LY1 and OCI-LY7 cell lines. Total RNA was extracted followed by cDNA synthesis using oligo dT primers. Gene specific primers were used to amplify 4E-BP1, 4E-BP2 and actin in a 30 cycle PCR reaction. Data representative of 3 independent experiments.
(TIFF)

Figure S4 These graphs depict the separate renilla and firefly luciferase values for the experiments shown in Figure 3A. The graph on the lower right shows the average luciferase counts in MLN0128-treated cells relative to DMSO-treated cells, again separated into renilla and firefly luciferase.
(TIFF)

Figure S5 Quantitative -real time PCR of total RNA to assess MCL-1, Cyclin D3 protein and mRNA levels in VAL, OCI-LY1 and OCI-LY7 partially serum starved in 0.1% FBS for 24 hrs and treated with 100 nM MLN0128 in 10% FBS media for 4 hours. Results representative of 3 independent experiments.
(TIFF)

Figure S6 Confirmation of results in Figure 5, using 7-AAD staining to measure cell death. Induction of cell death by a 48 hour treatment of 100 nM MLN0128 in the VAL and OCI-LY1 cells with lentiviral mediated eIF4E shRNA knockdown. Results represent the percentage of cells with sub-diploid DNA content and are averaged for 5 different experiments. Statistical significance was measured using a student’s t-test (paired, two-tailed) with error bars representing SEM (*p<0.05, **p<0.01 ***p<0.001,****p<0.0001). Shown below the graphs are western blots depicting the efficiency of eIF4E knockdown compared to the scrambled shRNA controls.
(TIFF)

Figure S7 eIF4E knockdown in VAL cells results in greater MCL-1 downregulation following MLN0128 treatment.
(TIFF)

Figure S8 Results of Oncomine expression database analysis. The Basso Lymphoma microarray study was queried for expression of eIF4E. The specimens representing normal B cells of various types are shown in the green box. Specimens representing Burkitt’s Lymphoma are boxed in orange, and DLBCL in red.
(TIF)

Figure S9 The Basso Lymphoma microarray study was queried for expression of 4EBP1. The specimens representing normal B cells of various types are shown in the green box. Specimens representing Burkitt’s Lymphoma are boxed in orange, and DLBCL in red. The red arrow points to the DLBCL specimen with very low 4EBP1 expression.
(TIF)

Figure S10 The Basso Lymphoma microarray study was queried for expression of 4EBP2. The specimens representing normal B cells of various types are shown in the green box. Specimens representing Burkitt’s Lymphoma are boxed in orange, and DLBCL in red.
(TIF)
