# Supplementary figures and images for: Resistance to mTOR Kinase Inhibitors in Lymphoma Cells Lacking 4EBP1
Source: PLoS One. 2014 Feb 21;9(2):e88865. doi: 10.1371/journal.pone.0088865 (PMC3931643; doi:10.1371/journal.pone.0088865)

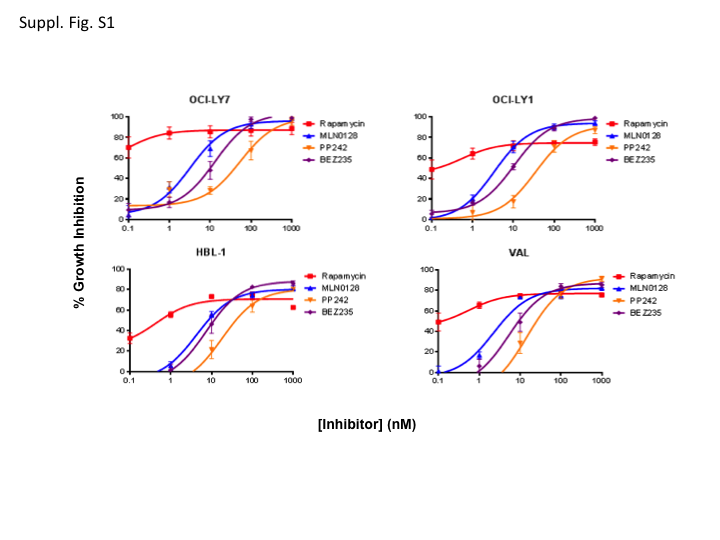

Supplement: Figure S1 — MTS assay to identify effective concentration ranges of mTOR inhibitors in DLBCL cell lines. Cells were treated with indicated concentrations of MLN0128, PP242, rapamycin or BEZ235 for 48 hours. Results were averaged from three independent experiments and plotted as percent growth inhibition. Error bars represent standard error of the mean (SEM). (TIFF) [file pone.0088865.s001.tiff]

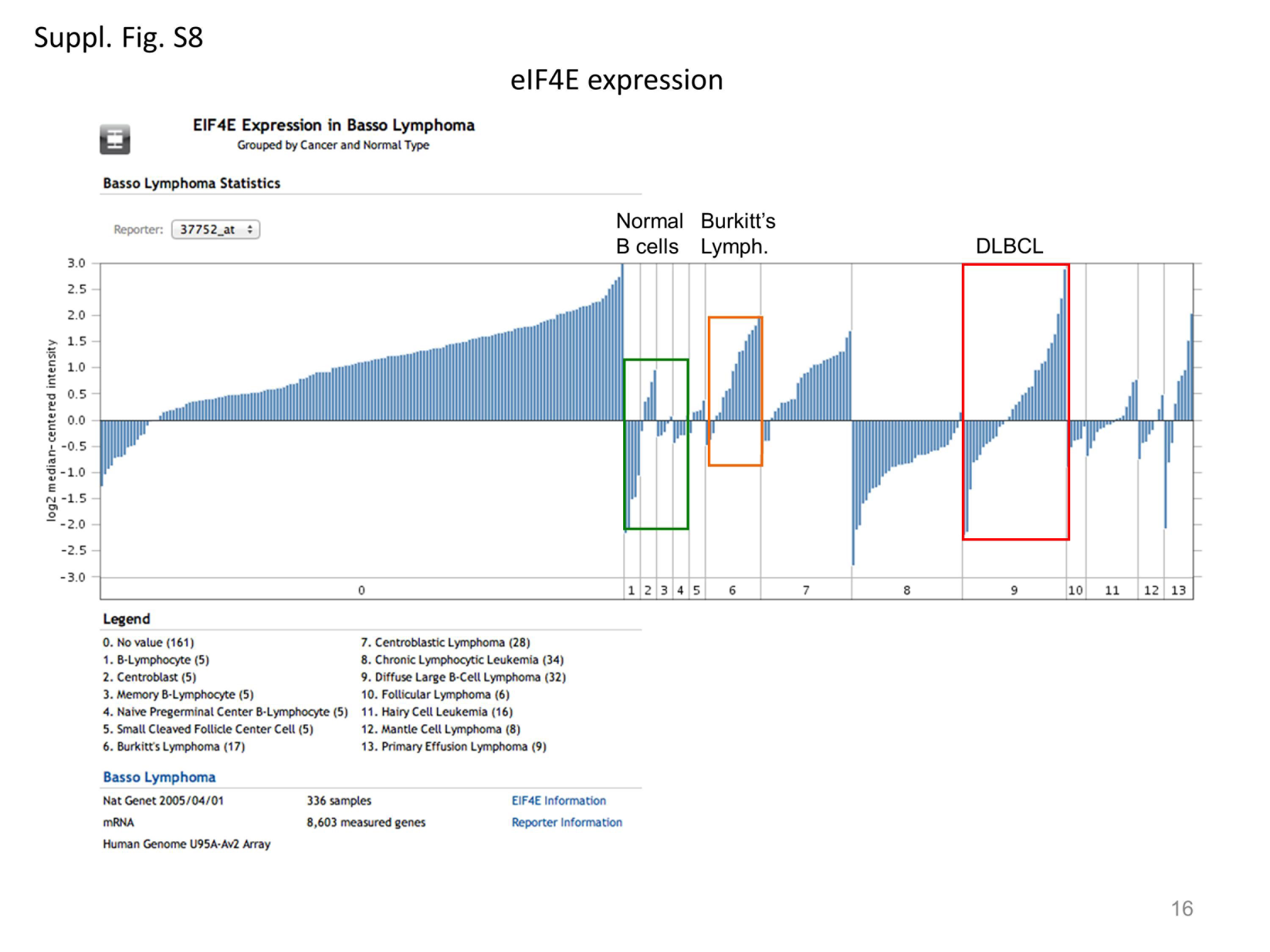

Supplement: Figure S8 — Results of Oncomine expression database analysis. The Basso Lymphoma microarray study was queried for expression of eIF4E. The specimens representing normal B cells of various types are shown in the green box. Specimens representing Burkitt’s Lymphoma are boxed in orange, and DLBCL in red. (TIF) [file pone.0088865.s008.tif]

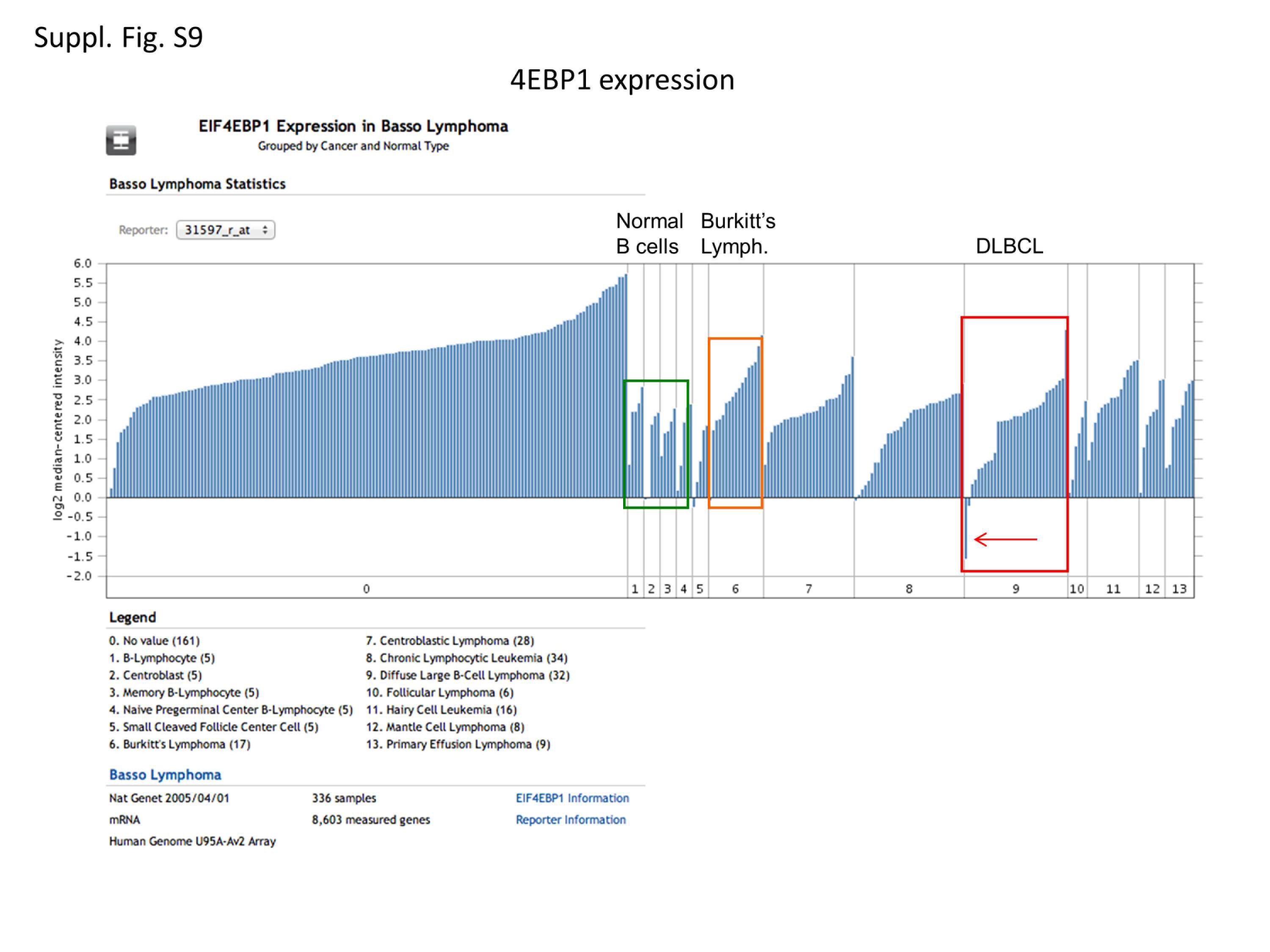

Supplement: Figure S9 — The Basso Lymphoma microarray study was queried for expression of 4EBP1. The specimens representing normal B cells of various types are shown in the green box. Specimens representing Burkitt’s Lymphoma are boxed in orange, and DLBCL in red. The red arrow points to the DLBCL specimen with very low 4EBP1 expression. (TIF) [file pone.0088865.s009.tif]

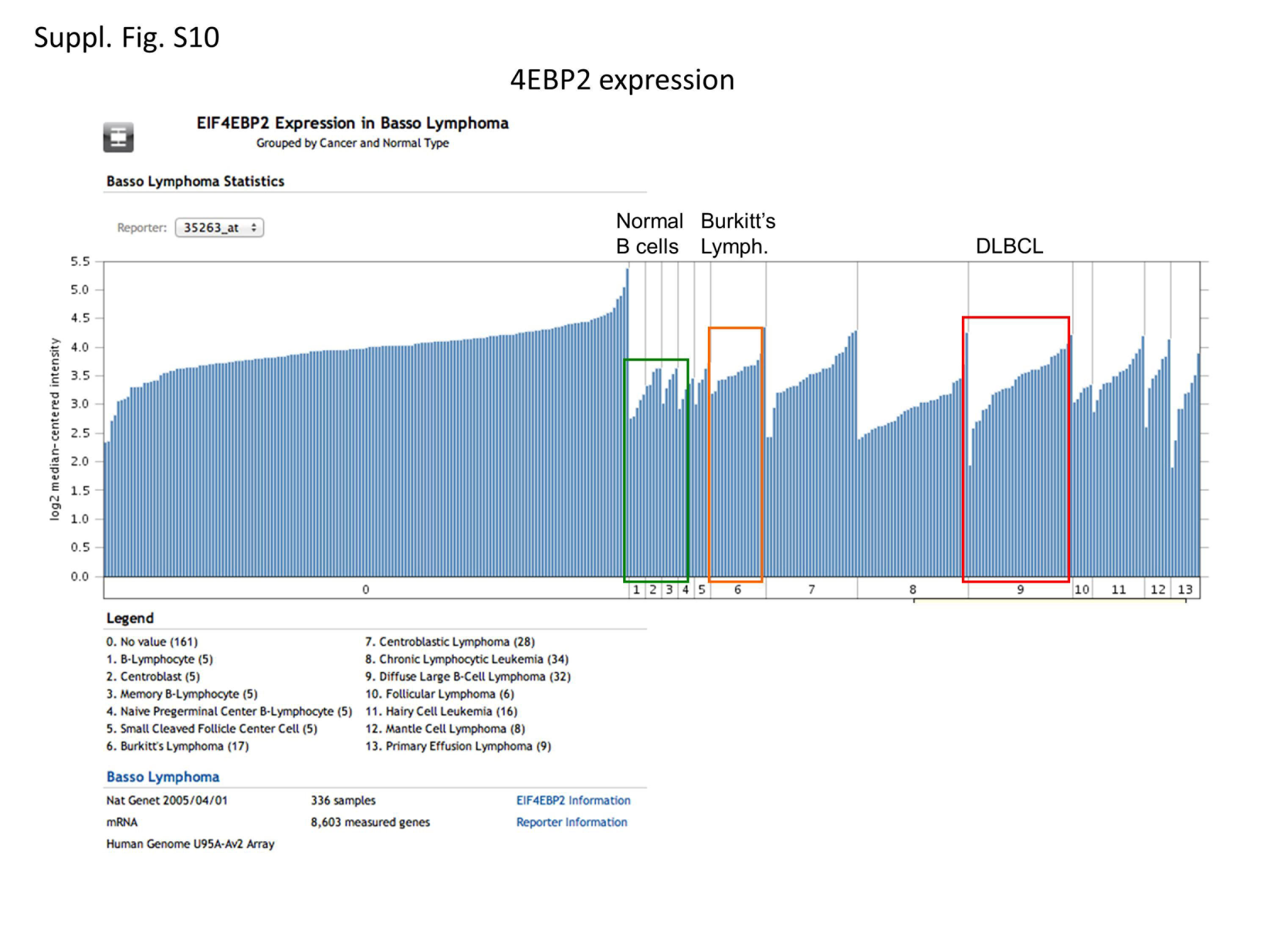

Supplement: Figure S10 — The Basso Lymphoma microarray study was queried for expression of 4EBP2. The specimens representing normal B cells of various types are shown in the green box. Specimens representing Burkitt’s Lymphoma are boxed in orange, and DLBCL in red. (TIF) [file pone.0088865.s010.tif]
